# Improvement of Physical Functions in Elderly Patients with Heart Failure Depends on the Hepatic Reserve

**DOI:** 10.1298/ptr.E10328

**Published:** 2025-03-10

**Authors:** Daisuke KUWAHARA, Takuya UMEHARA, Nobuhiro KITO

**Affiliations:** 1 Department of Rehabilitation, Saiseikai Kure Hospital, Japan; 2 Department of Rehabilitation, Faculty of Rehabilitation, Hiroshima International University, Japan

**Keywords:** Aged, Heart failure, Liver dysfunction, Physical function, Exercise

## Abstract

Objectives: In recent years, the number of elderly heart failure patients with multiorgan failure has been increasing. Furthermore, the combination of heart failure and decreased hepatic reserve can cause severe skeletal muscle impairment and decreased survival rates. This study investigated whether the degree of improvement in the five repetitions of sit-to-stand (5STS) and walking speed (WS) differs depending on hepatic reserve in elderly heart failure patients. Methods: The patients were divided into the following two groups: good hepatic reserve (albumin–bilirubin score [ALBI score] ≤−2.25) and poor hepatic reserve (ALBI score >−2.25). Propensity score matching was performed using the brain natriuretic peptide level. A two-way analysis of variance (ANOVA) was performed to examine the main effects of the hepatic reserve and time points (admission or discharge). Results: After propensity score matching, 28 out of the 33 (84.8%) patients in the good hepatic reserve (age, 83.74 ± 9.25 years and ALBI score, −2.55 ± 0.19 points) and 27 out of 40 (67.5%)patients in the poor hepatic reserve (age, 85.85 ± 7.53 years and ALBI score, −1.93 ± 0.26 points) were analyzed. Two-way ANOVA showed that the 5STS (p = 0.04) and WS (p = 0.01) in poor hepatic reserve tended to be worse than in good hepatic reserve. Furthermore, the 5STS (p = 0.04) and WS tended to improve at discharge in both groups. However, the improvement in WS was not significant (p = 0.15). Conclusions: Our study suggests that the hepatic reserve in elderly heart failure patients may be an important factor in the assessment of physical functions.

## Introduction

The incidence of elderly heart failure patients is increasing worldwide, and they are predicted that >300000 will be detected annually between 2020 and 2030^1)^. Elderly heart failure patients often have multiple organ dysfunctions, such as renal impairment and decreased hepatic reserve^2)^. In particular, the combination of heart failure and decreased hepatic reserve in elderly patients can cause cardio-hepatic-skeletal muscle syndrome, resulting in serious skeletal muscle impairment^3)^. The skeletal muscle impairment characteristic of heart failure patients includes a decrease in slow muscle fibers and aerobic metabolic function^4,5)^, which decreases the fundamental activities of daily living (ADL), such as chair sit to stand and walking ability^6–8)^. Furthermore, elderly patients with heart failure and decreased hepatic reserve are more likely to exhibit impairments in chair sit to stand and walking than those with heart failure only. Because decreased sit to stand time and walking speed (WS) are associated with decreased survival rates in heart failure and liver disease^3,9–11)^, these parameters should be improved as early as possible.

In the cross-sectional study by Noda et al.^3)^, the grip strength, isometric knee extension strength, and WS in elderly heart failure patients with decreased hepatic reserve were worse than those without decreased hepatic reserve. Furthermore, the hepatic reserve in elderly heart failure patients is associated with lower grip strength, isometric knee extension strength, and WS as much as or even more than cardiac function^3)^. However, no study has determined whether the sit-to-stand time and WS can be improved in elderly patients with heart failure and decreased hepatic reserve. A systematic review by Kuwahara et al.^12)^, and Zhang et al.^13)^, reported that exercise therapy can improve the chair sit-to-stand time and WS in elderly heart failure patients as well as those with decreased hepatic reserve. Therefore, even elderly patients with heart failure and decreased hepatic reserve may be able to improve their chair sit to stand time and WS.

The purpose of this study was to investigate whether the degree of improvement in chair sit-to-stand time and WS differs depending on whether there is decreased hepatic reserve in elderly heart failure patients. The results of this study will provide useful insights into determining rehabilitation strategies for hospitalized elderly heart failure patients.

## Methods

### Study design

This retrospective cohort study was conducted in accordance with the principles of the Declaration of Helsinki, and it was approved by the Hospital’s Ethics Committee of Saiseikai Kure Hospital (Approval number 155). Patients or their representatives were offered the opportunity to refuse participation via an opt-out format.

### Setting

The heart failure patients were enrolled between June 2021 and October 2022, and data were collected by physical therapists or occupational therapists.

### Patients

The patients with heart failure were hospitalized and treated for either acute heart failure or acute exacerbation of chronic heart failure. The definition of heart failure was based on the Framingham criteria^14)^. The inclusion criteria in heart failure patients were as follows: (a) those aged ≥ 65 years, (b) those who could walk independently before admission, and (c) those who do not show signs of worsening heart failure. The exclusion criteria in heart failure patients were as follows: (a) those who were discharged due to worsening of heart failure or other complications, (b) those who died during hospitalization, (c) those who had severe dementia (defined as Hasegawa Dementia Scale-Revised (HDS-R)score of ≤ 9)^15)^, and (d) those who had missing data. Based on the albumin–bilirubin score (ALBI) score, patients were divided into the following two groups: good hepatic reserve (ALBI score ≤ −2.25) and poor hepatic reserve (ALBI score > −2.25)^16)^. The ALBI score is calculated using the following formula: (log10 total bilirubin [mmol/L] × 0.66) + (albumin [g/L] × −0.085). Higher ALBI scores indicated poorer hepatic reserve capacity. The ALBI score exhibits good criterion-related validity with the Child-Pugh classification, which is the gold standard for assessing hepatic reserve capacity^17)^.

### Measurements

The following patient data were collected at the time of admission: age, sex, body mass index (BMI), brain natriuretic peptide (BNP) level, estimated glomerular filtration rate (eGFR), hemoglobin level, albumin level, total bilirubin level, New York Heart Association classification (NYHA), medical history (presence or absence of heart failure, stroke, and chronic kidney disease), life space assessment (LSA) score^18)^, length of hospital stay, HDS-R score, and physical frailty assessment index using the Japanese version of the Cardiovascular Health Study criteria (J-CHS)^19)^. The LSA is an indicator that quantifies activity in the past month across five spaces: indoors, on the premises, in the neighborhood, in town, and out of town. The LSA score ranges from 0 to 120, with higher scores indicating greater activity^18)^. The J-CHS consists of five items: weight loss, muscle weakness, tiredness, decreased WS, and decreased physical activity. Patients with none of the five components were considered non-frail (robust). Patients with only one or two components were considered prefrail, while those with three or more components were considered frail^19)^.

### Physical functions

Physical functions were assessed via WS and five repetitions of the sit to stand (5STS) test according to the procedures outlined in the Short Physical Performance Battery (SPPB)^20)^. The maximum WS over 4 m without rest was measured. The use of walking aids was optional, and the faster value of two measurements was recorded. In the 5STS test, the time taken to stand up five times from a seated position with their arms crossed over their chest was measured. The chair height was standardized at 45 cm to avoid differences between patients. The WS and 5STS were scored using the SPPB, which is a validated measure of physical function in elderly heart failure patients^20)^. Physical functions were assessed at the time of admission and discharge.

### Inpatient cardiac rehabilitation intervention program

Cardiac rehabilitation interventions for heart failure patients were performed to improve deconditioning and expand ADL during hospitalization. Cardiac rehabilitation interventions consisted of patient education and exercise therapy. Patient education during hospitalization included lifestyle advice on nutrition, medication use, and weight management. The exercise program included warm-up exercises, aerobic exercises such as walking or using a bicycle ergometer, and muscle-strength exercises, delivered as 40- to 80-minute sessions five times a week of exercise therapy. The exercise intensity was set at 30%–50% of heart rate reserve or a perceived exertion rate of 12–14 on a scale of 20. The type of exercise therapy was gradually changed, the duration of exercise was extended, and the load was increased according to the patient’s condition. Cardiac rehabilitation programs did not distinguish between the two groups: those with good hepatic reserve and those with poor hepatic reserve.

### Statistical analysis

All statistical analyses were performed using IBM SPSS Statics (ver. 29 IBM Japan, Tokyo, Japan). The significance level was set at 0.05. It is known that a decrease in WS and lower limb muscle strength is caused by decreased cardiac or/and hepatic function^21–23)^. Therefore, this study matched BNP, an indicator of heart function, as a propensity score to clarify whether the difference in WS and 5STS were attributable to hepatic reserve^24)^. To assess confounding factor adjustment, we compared patient characteristics between the good and poor hepatic reserve groups after propensity score matching. The characteristics of the patients in the good and poor hepatic reserve groups were compared using the unpaired t-test, Mann–Whitney’s U-test, or chi-square test. The WS and 5STS were assessed using a two-way analysis of variance (ANOVA) to examine the main effects and interaction of hepatic reserve (good or poor) and time point (admission or discharge). If significant main effects or interactions were detected, post hoc analysis was performed using the Bonferroni test.

### Sample size

The study size was calculated using MedCalc (version 19.2; MedCalc Software, Ostend, Belgium). The good and poor hepatic reserve groups (positive/negative ratio) varied between previous study^16)^. Therefore, a positive/negative ratio of 3:4 was used in this study. In this study, the alpha, statistical power, and area under the curve were set at 0.05, 0.80, and 0.7, respectively, to indicate the superiority of statistical discrimination. It was determined that a sample size of 73 patients was required, with 33 in the good hepatic reserve group and 40 in the poor hepatic reserve group.

## Results

### Characteristics of the patients in the good and poor hepatic reserve groups

**Fig. 1.**
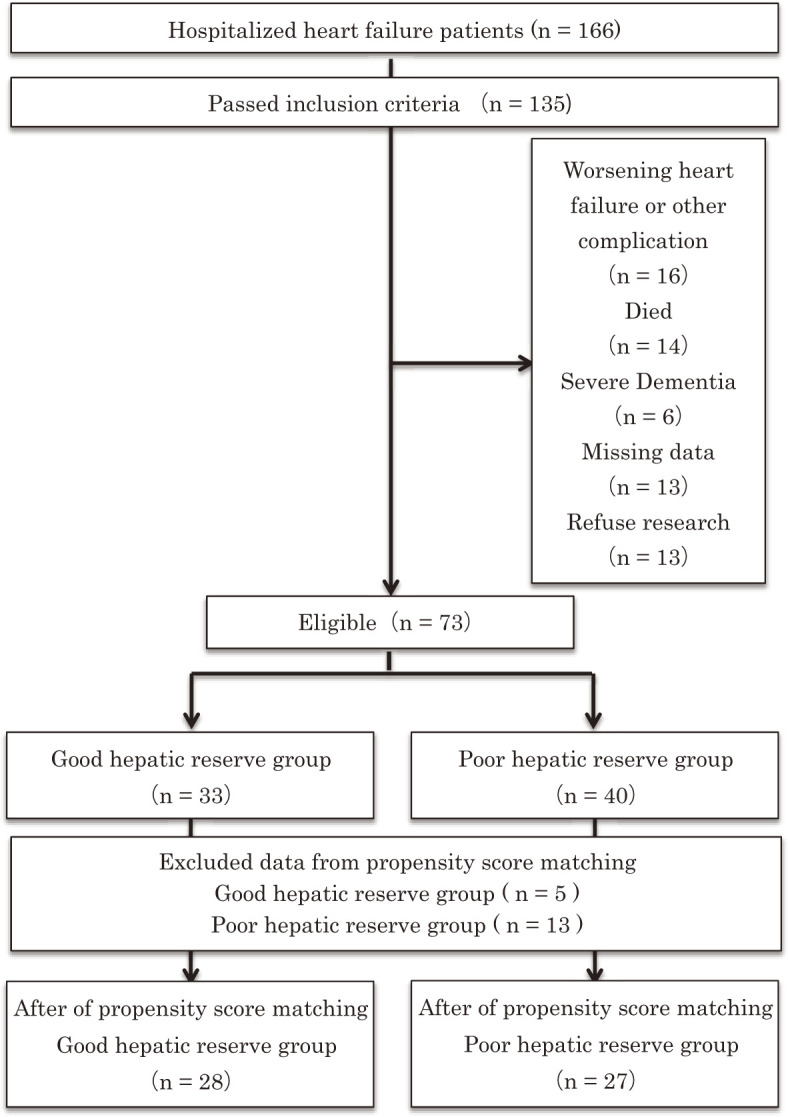
Flowchart for selecting patients

Figure 1 shows a flowchart of the patient selection process. A total of 73 patients met the eligibility criteria, with 33 in the good hepatic reserve group and 40 in the poor hepatic reserve group. After propensity score matching, 28 patients (84.8%) in the good hepatic reserve group and 27 patients (67.5%) in the poor hepatic reserve group were analyzed. Table 1 shows the patient characteristics in each group before propensity score matching. Before propensity score matching, the BNP (p = 0.03), hemoglobin (p = 0.03), total bilirubin (p = 0.03), and albumin (p = 0.00) levels were significantly lower and the ALBI score (p = 0.00), ALBI grade (p = 0.00), and LSA score (p = 0.04) were significantly poorer in the poor hepatic reserve group than in the good hepatic reserve group. Table 2 shows the patient characteristics in each group after propensity score matching. After propensity score matching, the hemoglobin (p = 0.02) and albumin (p = 0.00) levels were significantly lower and the ALBI score (p = 0.00), ALBI grade (p = 0.00), and LSA score (p = 0.03) were significantly poorer in the poor hepatic reserve group than in the good hepatic reserve group. The differences in the other variables were not significant between the two groups.

**Table 1.** Characteristics of patients in good and poor hepatic reserve (before propensity score)

Variables	Good hepatic reserve group (n = 33)	Poor hepatic reserve group (n = 40)	p Value
Age (years)	84.03 ± 8.82	86.00 ± 6.94	0.43^†^
Sex (female, n(%))	13 (39.39)	16 (40.00)	0.58^*^
BMI (kg/m^2^)	21.16 ± 3.57	21.12 ± 4.03	0.96^‡^
NYHA classification (I/II/III/IV)	5/20/8/0	5/19/16/0	0.37^*^
Presence of medical history, n (%)			
Heart failure	23 (69.70)	31 (77.50)	0.31^*^
Stroke	10 (30.30)	6 (15.0)	0.09^*^
Chronic kidney disease	10 (30.30)	17 (42.50)	0.20^*^
BNP (pg/mL)	283.75 ± 477.87	503.04 ± 585.28	0.03^†^
eGFR (mL/min/1.73 m^2^)	41.76 ± 16.69	37.95 ± 18.76	0.33^‡^
Hemoglobin (g/dL)	12.73 ± 5.74	10.70 ± 2.16	0.03^†^
Albumin (g/dL)	3.75 ± 0.26	3.09 ± 0.43	0.00^†^
ALBI score (points)	−2.52 ± 0.20	−1.87 ± 0.33	0.00^†^
ALBI grade (I/IIa/IIb/III)	(9/23/1/0)	(0/0/35/5)	0.00^*^
Total bilirubin (mg/dL)	0.69 ± 0.46	0.97 ± 0.70	0.03^†^
Creatinine (mL/min/1.73m^2^)	1.25 ± 0.73	1.38 ± 0.64	0.26^†^
AST	22.36 ± 6.45	36.22 ± 47.46	0.04^†^
ALT	18.00 ± 15.27	28.45 ± 47.98	0.36^†^
Frail (robust/pre-frail/frail)	1/6/26	1/4/35	0.59^*^
LSA (points)	34.77 ± 28.31	26.46 ± 30.23	0.04^†^
Length of stay (days)	43.27 ± 21.98	44.20 ± 17.33	0.84^‡^
HDS-R (points)	21.90 ± 5.33	17.97 ± 7.87	0.08^†^
Medication, n (%)			
Dopamine	0 (0.00)	0 (0.00)	N.A.
Norepinephrine	0 (0.00)	0 (0.00)	N.A.
Dobutamine	0 (0.00)	0 (0.00)	N.A.
PDE-III inhibitor	7 (21.21)	7 (17.50)	0.77*
Carperitide	1 (3.03)	4 (10.00)	0.25*
Diuretic	23 (69.70)	31 (77.50)	0.31*
Beta-blocker	15 (45.45)	19 (47.50)	0.53*

Value: average ± standard deviation.

^†^Mann–Whitney U test, ^‡^unpaired t test, *χ^2^ test.

BMI, body mass index; NYHA, New York Heart Association; BNP, brain natriuretic peptide; eGFR, estimated glomerular filtration rate; ALBI, albumin–bilirubin; AST, aspartate aminotransferase; ALT, alanine aminotransferase; LSA, life space assessment; HDS-R, Hasegawa Dementia Scale-Revised; PDE, phosphodiesterase

**Table 2.** Characteristics of patients in good and poor hepatic reserve (after propensity score)

Variables	Good hepatic reserve group (n = 28)	Poor hepatic reserve group (n = 27)	p Value
Age (years)	83.74 ± 9.25	85.85 ± 7.53	0.37^†^
Sex (female, n (%))	10 (35.71)	13 (46.43)	0.29^*^
BMI (kg/m^2^)	21.14 ± 3.71	21.39 ± 3.91	0.81^‡^
NYHA classification (I/II/III/IV)	4/15/9/0	4/14/9/0	0.96^*^
Presence of medical history, n (%)			
Heart failure	19 (67.86)	21 (75.00)	0.38^*^
Stroke	8 (28.57)	5 (17.86)	0.26^*^
Chronic kidney disease	8 (28.57)	10 (35.71)	0.38^*^
BNP (pg/ml)	300.59 ± 508.1	300.62 ± 440.12	0.96^†^
eGFR (mL/min/1.73 m^2^)	42.66 ± 16.82	38.89 ± 17.89	0.42^‡^
Hemoglobin (g/dL)	13.14 ± 6.27	10.87 ± 1.83	0.02^‡^
Albumin (g/dL)	3.80 ± 0.25	3.12 ± 0.43	0.00^‡^
ALBI score(points)	–2.55 ± 0.19	–1.93 ± 0.26	0.00^†^
ALBI grade (I/IIa/IIb/III)	(9/18/1/0)	(0/0/25/2)	0.00^*^
Total bilirubin (mg/dL)	0.68 ± 0.48	0.88 ± 0.66	0.10^†^
Creatinine (ml/min/1.73m^2^)	1.23 ± 0.70	1.41 ± 0.62	0.11^†^
AST	22.92 ± 6.75	32.03 ± 28.92	0.09^†^
ALT	18.96 ± 16.60	25.11 ± 37.03	0.44^†^
Frail (robust/pre-frail/frail)	1/2/25	1/4/22	0.70*
LSA (points)	34.05 ± 25.72	26.08 ± 29.41	0.03^†^
Length of stay (days)	44.00 ± 22.74	46.68 ± 15.57	0.60^‡^
HDS-R (points)	21.34 ± 5.49	17.07 ± 7.94	0.06^†^
Medication (%)			
Dopamine	0 (0.00)	0 (0.00)	N.A.
Norepinephrine	0 (0.00)	0 (0.00)	N.A.
Dobutamine	0 (0.00)	0 (0.00)	N.A.
PDE-III inhibitor	5 (17.86)	5 (17.86)	0.63*
Carperitide	1 (3.57)	3 (10.71)	0.30*
Diuretic	20 (71.43)	23 (82.14)	0.26*
Beta-blocker	14 (50.00)	16 (57.14)	0.39*

Value: average ± standard deviation.

^†^Mann–Whitney U test, ^‡^Unpaired t test, *χ^2^ test.

BMI, body mass index; NYHA, New York Heart Association; BNP, brain natriuretic peptide; eGFR, estimated glomerular filtration rate; ALBI, albumin–bilirubin; AST, aspartate aminotransferase; ALT, alanine aminotransferase; LSA, life space assessment; HDS-R, Hasegawa Dementia Scale-Revised; PDE, phosphodiesterase

### Differences in hepatic reserve and time of 5STS and WS before and after propensity score matching

Table 3 shows differences in hepatic reserve and time of 5STS and WS before propensity score matching. Before propensity score matching, 5STS at admission tended to improve at discharge, but the difference was not significant (good hepatic reserve group: p = 0.34, poor hepatic reserve group: p = 0.29). The WS in the poor hepatic reserve group tended to be worse than the good hepatic reserve group, but the difference was not significant (admission: p = 0.34, discharge: p = 0.29). Before propensity score matching, there were no significant differences in 5STS and WS between the other groups and times. Table 4 shows differences in hepatic reserve and time of 5STS and WS after propensity score matching. After propensity score matching, 5STS at admission tended to improve at discharge, but the difference was not significant (good hepatic reserve group: p = 0.21, poor hepatic reserve group: p = 0.11). The 5STS and WS in the poor hepatic reserve group tended to be worse than good hepatic reserve group, but the differences were not significant (5STS admission: p = 0.11, discharge: p = 0.21. WS admission: p = 0.07, discharge: p = 0.06). After propensity score matching, there were no significant differences in 5STS and WS between the other groups and times.

**Table 3.** Two-way ANOVA results for 5STS and WS (before propensity score)

	Good hepatic reserve group (n = 33)		Poor hepatic reserve group (n = 40)		Main effect Interaction
	Admission	Discharge	Admission	Discharge	
5 STS	1.12 ± 1.39	1.59 ± 1.37	0.72 ± 1.03	1.20 ± 1.36	Hepatic reserve : p = 0.06 Time point : p = 0.02^*^ Hepatic × time : p = 0.98
WS	2.58 ± 1.09	2.84 ± 1.14	2.08 ± 1.16	2.35 ± 1.15	Hepatic reserve : p = 0.01^*^ Time point : p = 0.15 Hepatic × time : p = 0.98

Value: average ± standard deviation.

*p <0.01.

ANOVA, analysis of variance; 5STS, five repetition of sit to stand; WS, walking speed

**Table 4.** Two-way ANOVA results for 5STS and WS (after propensity score)

	Good hepatic reserve group (n = 28)		Poor hepatic reserve group (n = 27)		Main effect Interaction
	Admission	Discharge	Admission	Discharge	
5 STS	1.25 ± 1.46	1.70 ± 1.38	0.67 ± 1.03	1.25 ± 1.40	Hepatic reserve : p = 0.04^*^ Time point : p = 0.04^*^ Hepatic × time : p = 0.79
WS	2.61 ± 1.10	2.93 ± 1.07	2.07 ± 1.07	2.36 ± 1.12	Hepatic reserve : p = 0.01^*^ Time point : p = 0.15 Hepatic × time : p = 0.93

Value: average ± standard deviation.

*p < 0.01.

ANOVA, analysis of variance; 5STS, five repetition of sit to stand; WS, walking speed

## Discussion

### Summary

The purpose of this study was to investigate whether the degree of improvement in chair sit-to-stand time and WS differs depending on whether there is decreased hepatic reserve in heart failure patients. Our study revealed that the 5STS and WS tended to be worse in the poor hepatic reserve group than in the good hepatic reserve group. Furthermore, both groups tended to improve in 5STS and WS from admission to discharge. Thus, rehabilitation during hospitalization could lead to improvement of 5STS and WS.

### Difference in the 5STS between the two hepatic reserve groups

After propensity score matching, the 5STS tended to be worse in the poor hepatic reserve group than in the good hepatic reserve group. However, this difference was not statistically significant. There have been no reports examining whether the 5STS was worse in heart failure patients with poor hepatic reserve compared to those without. We hypothesize that the tendency for 5STS to be worse in the poor hepatic reserve group than in the good hepatic reserve group was related to malnutrition and decreased hemoglobin. The decrease in hepatic reserve leads to malnutrition and decreased hemoglobin levels, resulting in muscle weakness^25–30)^. Furthermore, the decrease in hepatic reserve leads to impaired clearance of toxic substances in the hepatic^31)^. As a result, toxic substances are processed not only in the hepatic but also in skeletal muscles^32,33)^. However, it is known that during this process, the proteins necessary for exerting muscle strength are lost^31,32)^. The representative data for a protein is albumin. Albumin is a protein that is produced in the hepatic and is an indicator of nutritional status^34)^. Albumin levels are significantly negatively correlated (r = −0.5) with the 5STS in the elderly^35)^. In our study, the albumin level was significantly lower in the poor hepatic reserve group than in the good hepatic reserve group (3.80 ± 0.20 vs. 3.10 ± 0.40 g/dL). An albumin level of < 3.50 g/dL is indicative of malnutrition^36)^. Furthermore, the decrease in hepatic reserve is known to lead to a decrease in hemoglobin levels due to erythrocyte dysplasia and differentiation^29)^. The decrease in hemoglobin leads to a decrease in oxygen transport throughout the body and the aerobic energy metabolism required for sustained muscle contraction^37,38)^. In the 5STS, muscular endurance is required for sustained muscle contraction^39,40)^. Therefore, patients with low hemoglobin levels are likely to have low 5STS. In our study, the hemoglobin level was significantly lower in the poor hepatic reserve group than in the good hepatic reserve group (13.14 ± 0.2 vs. 10.87 ± 1.83 g/dL). The hemoglobin level in the poor hepatic reserve group was lower than the reference value for anemia (12.0 g/dL)^41)^. In a previous study, lower hemoglobin levels were significantly associated with lower 5STS in the elderly^42)^. Thus, the decrease in hepatic reserve capacity is associated with decreased albumin and hemoglobin levels, which decrease the muscle strength.

### Differences in 5STS between admission and discharge

After propensity score matching, the 5STS had improved at the time of discharge. However, this difference was not significant. A previous systematic review and meta-analysis^12)^ determined that the 5STS in heart failure patients improves with inpatient exercise therapy. In our study, the 5STS improved even in elderly heart failure with decreased hepatic reserve. Thus, we hypothesized that exercise therapy and severity of hepatic reserve may affect the 5STS. If impairment of hepatic reserve can be compensated, the endocrine action of skeletal muscles during exercise therapy can improve muscle strength and hepatic reserve^43–46)^. However, if the impairment of hepatic reserve is severe, hepatic-derived transmitters could inhibit the effects of exercise therapy on muscle strength. An ALBI grade of II or lower is considered as compensated impairment of hepatic reserve^17)^. In our study, the proportion of patients with ALBI grade II was 92.6% in the poor hepatic reserve group and 100.0% in the good hepatic reserve group. This indicates that patients more likely to experience improved lower limb muscle strength with exercise therapy may have been selected in the study.

### Difference in the WS between the poor and good hepatic reserve groups

After propensity score matching, the WS tended to be worse in the poor hepatic reserve group than in the good hepatic reserve group. However, this difference was not significant. This finding is consistent with those of previous studies that have demonstrated that elderly patients with heart failure and poor hepatic reserve have a worse WS than elderly patients with heart failure and good hepatic reserve^3)^. Thus, muscle strength is lower in patients with poor hepatic reserves than in those with good hepatic reserves. Muscle strength and mass, such as grip strength and skeletal muscle mass, are considered important determinants of WS in the elderly. Both grip strength (male: r = 0.52, female: r = 0.52) and limb skeletal muscle mass (male: r = 0.31, female: r = 0.51) showed significant positive correlation with WS^47)^. However, it has been reported that there is no significant correlation between limb skeletal muscle mass and WS in elderly patients of the same age group with hepatic disease^47)^. Furthermore, it has also been found that the correlation between grip strength and WS decreases compared to the elderly of the same age (male: r = 0.37, female: r = 0.39)^47)^. Additionally, factors other than muscle weakness may be involved in the decrease in WS in elderly patients with poor hepatic reserves^47)^. One of these factors is the activity level. Decreased activity due to a poor hepatic reserve capacity results from impaired energy storage and metabolism as well as impaired clearance of toxic substances^48)^. In our study, among the patients with heart failure, those with a poor hepatic reserve had a significantly worse LSA than those with a good hepatic reserve. Similarly, a previous study also reported that LSA, a measure of physical activity in elderly people of the same age as in this study, correlated as well with WS (r = −0.44) as sarcopenia severity (r = −0.42), a measure of muscle mass and muscle weakness^49)^. Thus, the deterioration of WS in elderly patients with poor hepatic reserves may be caused by decreased muscle strength and physical activity.

### Differences in WS between admission and discharge

After propensity score matching, there was no significant difference in the WS between the values at admission and discharge. There have no reports examining whether WS improves during hospitalization in elderly heart failure patients with poor hepatic reserve. We believed that although the WS in this study did not improve during hospitalization, there were important changes. The WS in this study improved by about 0.3 points during the hospitalization in both the good and poor hepatic reserve groups: this study scored WS based on the SPPB. In elderly people in the same age group, the 95% confidence interval for the minimal clinically important difference (MCID95) in SPPB due to exercise therapy ranged from 0.3 to 0.8 points^50)^. In other words, even in elderly heart failure patients, regardless of the poor hepatic reserve, improvement in WS equivalent to the MCID95 of the same age group was observed. This may explain the improvement in 5STS. Muscle strength is significantly related to WS in patients of the same age and with the same degree of poor hepatic reserve as that in the present study^51)^. Furthermore, WS is significantly negatively correlated with 5STS (r = −0.51) in elderly heart failure patients^52)^. These reports^51,52)^ suggest that, among heart failure patients, those with poor hepatic reserve who improve their 5STS are also likely to improve their WS.

### Interpretation and clinical application of the study results

This study was the first report that the physical functions (i.e., 5STS and WS) in elderly heart failure patients were analyzed based on the presence or absence of decreased hepatic reserve. Many previous reports have revealed that heart failure and reduced hepatic reserve cause symptoms such as tachycardia and fatigue during exercise^53,54)^. We demonstrated that low-load and high-frequency exercise therapy might improve 5STS and WS in elderly heart failure patients with reduced hepatic reserve considering tachycardia and fatigue during exercise. However, 5STS and WS remained lower during hospitalization in patients with decreased hepatic reserve than in those without. Therefore, the presence or absence of decreased hepatic reserve might be an important evaluation index for assessing the status and prognosis based on 5STS and WS. The 5STS and WS used in this study were movement tasks that require balance ability and begin from a quiet sitting or standing position^55,56)^. The 5STS and WS were also important parameters that contributed to improving the ADL of elderly people^57,58)^. In particular, elderly heart failure patients in hospitals or decreased hepatic reserve often have limited activity due to their symptoms and the constrained living environment of the hospital. Therefore, the ability to stand up and walk was considered essential for improving ADL. Our results provided new insights that this study aimed at improving physical functions and ADL into reconsidering rehabilitation strategies in elderly heart failure patients.

### Limitations

This study had two major limitations. First, the average age of the patients was high (approximately 85 years). Age is associated with a decrease in cardiac function, hepatic reserve, and physical function^3,52–59)^. Therefore, the study results can only be applied to elderly heart failure patients. Furthermore, these results should be applied with caution in young heart failure patients. Second, the ALBI score, which is an indicator of hepatic reserve, was only determined at the time of admission. The hepatic reserve improves due to the exercise therapy-induced improvement in skeletal muscle^46)^. Therefore, the changes in 5STS and WS in this study may have been related to the effect of exercise therapy as well as improvement in hepatic reserve. In future studies, the ALBI score at discharge should be assessed.

In conclusion, this study’s results indicate that the hepatic reserve in elderly heart failure patients may be an important factor in the assessment of physical functions.

## Acknowledgments

We thank the patients and staff members who helped with this study.

## Funding

Not applicable.

## Conflicts of Interest

There are no conflicts of interest in this study.
